# Calendar

**DOI:** 10.1155/S1463924685000347

**Published:** 1985


Journal of Automatic Chemistry/*Journal of Clinical Laboratory Automation*, Volume 7, No. 3 (Jul–Sep 1985), pp. 158–159

### Calendar

### SEPTEMBER 1985

### Sixth European Congress of Clinical Chemistry

To be held from 1–5 September in Jerusalem, Israel.

*Further information from Dr E. Shafkir, Chairman of the Co-ordinating Board: Clinical Chemistry and Enzymology Meetings, P.O. Box 50006, Tel-Aviv, Israel.*

### EMAG 85

To be held from 2 to 5 September at the University of Newcastle-upon-Tyne, UK. Organized by the Electron Microscopy and Analysis Group of the Institute of Physics. This is the sixth EMAG meeting and three departures from the themes of previous conferences will be: a greater emphasis on applications and the use of image interpretation techniques; the introduction of posters on brief theoretical and experimental topics; an instruction course on advanced analytical techniques immediately following the conference and employing exhibition equipment and lecturers from the conference.

*Further information from the Institute of Physics, 47 Belgrave Square, London SW1X 8QX.*

### 2nd Congress of the International Society of Animal Clinical Biochemistry

To be held from 2–6 September in Jerusalem, Israel.

*Further information from Dr E. Shafkir (above).*

### 5th International Congress on Clinical Enzymology

To be held from 3–6 September in Jerusalem, Israel.

*Further information from Dr E. Shafkir (above).*

### Flow Analysis III

To be held from 5 to 8 September in Birmingham, UK.

*Further information from Dr A. M. G. Macdonald, Department of Chemistry, The University, PO Box 363, Birmingham B15 2TT, UK; or from the Royal Society of Chemistry, Burlington House, London W1V 0BN.*

### 14th X-Ray Spectrometry Conference

To be held from 9 to 13 September at Durham University. Organized by Philips Analytical and the University's Department of Geological Sciences, coverage includes advances in instrumentation for X-ray spectrometry and applications of the technique, methods for sample preparation and presentation, qualitative and semi-quantitative analysis, data handling techniques and X-ray fluorescence as a standard method.

*Further information from Margaret Courtney, Conference Secretary, Philips Analytical Department, Pye Unicam Ltd, York Street, Cambridge CB1 2PX, UK.*

### IUPAC 1985

To be held from 9 to 13 September 1985 in Manchester, UK.

*Further information from the Royal Society of Chemistry, Burlington House, London W1V 0BN.*

### Third Asian-Pacific Congress of Clinical Biochemistry

To be held from 15 to 20 September in Bali. Organized by the Indonesian Association for Clinical Chemistry and the Pacific Federation of Clinical Biochemistry; symposia include: Computers in clinical laboratory management, Immunoassay, and Separation techniques.

*Further information from Professor R. Gandasoebrata, 11 Jl. Rajasa I, Blok R, Kebayoran Baru, Kakarta Selatan, Indonesia.*

### Colloquium Spectroscopicum Internationale XXIV

To be held from 15 to 21 September in Garmisch-Partenkirchen, FR Germany.

*Further information from CSI XXIV, Organisationsbüro, Institut für Spektrochemie und angewandte Spektroskopie, Postfach 778, D4600 Dortmund 1, FR Germany.*

### The British Laboratory Week, incorporating The Laboratory; Analyticon Exhibition & Conference; International Environment & Safety Exhibition and Conference; and The Medical Laboratory Sciences Exhibition

To be held from 16 to 19 September at Olympia in London. Sponsoring organizations include: the Royal Society of Chemistry, the Scientific Instrument Manufacturers' Association, the Chromatographic Society, the British Laboratory Ware Association, the Association of Clinical Biochemists and the Institute of Science Technology.

*Further information from Curtis/Steadman and Partners Ltd, The Hub, Emson Close, Saffron Walden, Essex CB10 1HL, UK. Tel: 0799 26699. Details about the International Environ-*

*ment & Safety Meetings from Diane Parker, 'Newgate', Sandpit Lane, St Albans, Hertfordshire AL4 0BS, UK.*

---

**The Automated Analysis of Ions in Solution**

To be held from 25 to 27 September at the University of York, UK.

*Further information from Dr Clive Jackson, Health & Safety Executive, 403 Edgware Road, London NW2 6LN.*

---

**12th Annual Meeting of the Federation of Analytical Chemistry and Spectroscopy Societies**

To be held from 29 September to 4 October in Philadelphia, Pennsylvania, USA.

*Further information from Allan Ullman, Proctor & Gamble Company, 6250 Center Hill Road, Cincinnati, Ohio 45224, USA.*

---

**Sixth Colloquium on Prospective Biology**

To be held from 30 September to 4 October at Pont-à-Mousson, France.

*Further information from Colloque Biologie Prospective, 30 rue Lionnois, BP 292, 54005 Nancy Cedex, France.*

---

### OCTOBER 1985

**35th Canadian Chemical Engineering Conference**

To be held from 6 to 9 October at the Calgary Convention Centre, Canada.

*Further information from Dr Roger M. Butler, Department of Chemical and Petroleum Engineering, University of Calgary, Calgary, Alberta T2N 1N4, Canada.*

---

**The Integrated Approach to Laboratory Automation: a review of analytical instrumentation, robotics, laboratory information management systems and laboratory design and staffing**

To be held from 23 to 25 October at the Dormy Hotel, Ferndown, Dorset, UK.

*Further information from Dr Clive Jackson, Health & Safety Executive, 403 Edgware Road, London NW2 6LN.*

---

**Pollution Monitoring & Control**

To be held from 29 to 31 October at Wembley, London.

*Further information from Trident International Exhibitions Ltd, 21 Plymouth Road, Tavistock, Devon PL19 8AU, UK.*

---

**Seminar – Laboratory Information Management**

To be held in October, probably in High Wycombe, Buckinghamshire, UK. Organized by Beckman, subjects to be included are CALS—a comprehensive sample tracking system for the analytical laboratory; practical benefits of CALS in the pharmaceutical Q/C laboratory; GLP compliance features of the CALS system; stability studies for the food and pharmaceutical industries; instrument calibration and maintenance software and system suitability programme for CALS. Practical demonstrations of the CALS system will also be part of the programme.

*Further information from John Boother, Beckman, Ltd, Progress Road, Sands Industrial Estate, High Wycombe, Buckinghamshire, UK. Tel: 0494 41181.*

---

### NOVEMBER 1985

**1985 Eastern Analytical Symposium**

To be held at the New York Penta, USA from 19 to 22 November.

*Further information from Harvey Gold, Department of Chemistry, University of Delaware, Newark, Delaware, 19716, USA.*

### DECEMBER 1985

**Chem Asia 85**

To be held from 2–5 December in Singapore.

*Further information from Chatsworth House, 59 London Road, Twickenham TW1 3SZ, UK.*

---

### 1986 MEETINGS

**Fourth World Filtration Congress**

To be held from 22 to 25 April 1986 in Ostend, Belgium.

*Further information from the Secretariat of World Filtration Congress, IV, KVIV-Technologisch Instituut, Jan van Rijswijcklaan 58, B 2018 Antwerpen, Belgium.*

---

**Analytica 86**

To be held from 3 to 6 June 1986 at München, FR Germany.

*Further information from Münchener Messe-und Austellungsgesellschaft mbH, Messegelände, Postfach 12 10 09, D8000 München 12, FR Germany.*

---

**SAC 86 3rd BNASS: an International Conference on Analytical Chemistry**

To be held from 20–26 July 1986 at the University of Bristol, UK.

*Further information from The Secretary, Analytical Division, Royal Society of Chemistry, Burlington House, London W1V 0BN.*

---

*Conference organizers should send full details of their meeting for inclusion in 'Journal of Automatic Chemistry's' calendar at least three months before the event. Information to the Editor, Journal of Automatic Chemistry, Taylor & Francis Ltd, 4 John Street, London WC1N 2ET.*

